# Awareness, Willingness, and Concerns about Clinical Trial Participation among Iraqi Patients: A Cross-Sectional Study

**DOI:** 10.5334/gh.1536

**Published:** 2026-03-23

**Authors:** Zainab Atiyah Dakhil, Noor Ali Hasan, Sarah K. Hassan, Ridha S. Nazzal, Ahmed Sermed Al Sakini, Mohammed Saad Qasim, Mohammed Qays, Mohammed Dheyaa Marsool Marsool, Hasan Ali Farhan, Jose Leal, Michele Peters

**Affiliations:** 1 Ibn Al-Bitar Cardiac Centre, University of Baghdad/Al-Kindy College of Medicine, Baghdad, Iraq; 2 University of Baghdad/Al-Kindy College of Medicine, Baghdad, Iraq; 3 University of Baghdad/College of Medicine, Baghdad, Iraq; 4 Mayo Clinic, Arizona, USA; 5 University Of Oxford, Oxford, UK

**Keywords:** clinical trials, patient public involvement, attitudes, health literacy, altruism, Middle East

## Abstract

**Background:**

Equitable representation in clinical trials (CTs) is essential for the validity, generalizability, and ethical integrity of medical research. However, participation from low- and middle-income countries (LMICs) remains disproportionately low, particularly in the Middle East and North Africa (MENA) region. Iraq, despite a substantial disease burden, has minimal participation in global clinical research, and patient-level determinants of CT engagement remain largely unexplored. Understanding patient perceptions in these settings is essential to enhance recruitment in CTs.

**Purpose:**

To assess awareness, willingness, motivators, and concerns regarding CT participation among Iraqi patients, and to identify demographic and perceptual factors associated with willingness to participate.

**Methods:**

A multi-center, cross-sectional study was conducted using a validated 16-item interviewer-administered survey among patients attending five major teaching hospitals in Baghdad, Iraq, between October 2023 and February 2024. The survey assessed demographic characteristics, awareness of CTs, prior participation, perceived motivators and barriers, and willingness to participate under different trial scenarios (invasive, non-invasive, digital, and drug safety contexts). If a respondent reported lack of knowledge of the term ‘clinical trial,’ the investigator explained the meaning in simple Arabic to enable the respondent to participate. Descriptive statistics, univariate and multivariate analyses were performed to examine associations between participant characteristics and willingness to participate.

**Results:**

A total of 631 patients (mean age 41.7 ± 16.2 years; 60.1% women) with generally low educational attainment (30.1% primary education; 14.4% no formal education) participated. Awareness of CTs was extremely limited, as 90.6% of participants had never heard of CTs and only 1.1% reported prior participation. More than half (51.3%) expressed concerns regarding participation, with safety being the predominant concern (85.9%), followed by family obligations that could limit trial adherence (55.8%), and fears of being experimented upon. Despite these concerns, altruistic motivations were prominent, with advancing medical science (86.4%) and helping other patients (85.4%) cited most frequently. If invited to participate in a CT, 40.1% of respondents indicated willingness, whereas 51.3% would decline and 8.6% were uncertain. Willingness varied substantially by trial characteristics: only 28.8% were willing to participate in trials involving invasive procedures, compared with the 61.2% who would participate in non-invasive studies, including educational interventions, telemedicine, or digital applications. Perceived drug safety was a key determinant, with willingness increasing to 70.8% when investigational drugs had confirmed safety, but declining sharply when safety was uncertain, with 85.9% declining willingness. Overall, educational level, prior awareness of CTs, and safety-related concerns were strongly associated with willingness to participate. In multivariable analysis, higher education (college/postgraduate) was independently associated with greater willingness to participate (OR 2.06, 95% CI 1.15–3.68), whereas having concerns about CTs was associated with reduced willingness (OR 0.42, 95% CI 0.27–0.66).

**Conclusions:**

Iraqi patients demonstrate profound gaps in awareness of CTs and substantial safety-related concerns, yet exhibit strong altruistic motivations and openness to non-invasive and digital research models. These findings underscore the need for culturally tailored public education, transparent communication regarding trial safety, and innovative trial designs to enhance participation. Addressing these barriers is critical to improve equitable representation of LMIC populations in general and of the MENA region in particular in global clinical research, and to strengthen the external validity of CT evidence.

## Strength and limitations

- This is the first systematic investigation of Iraqi patients’ perceptions of clinical trial participation, addressing a significant evidence gap in LMICs.
- The large sample size and inclusion of participants from five major teaching hospitals enhance the reliability and diversity of responses.
- Use of face-to-face interviews helped reduce literacy-related barriers and improved response accuracy.
- The study was conducted in hospital-based settings, which may not fully capture the views of the general community, particularly rural or marginalized populations.
- The limited survey length (16 items) and lack of data on participants’ health conditions may have restricted insight into disease-specific attitudes toward trial participation.

The key findings of awareness, willingness, and concerns to clinical trial participation are summarized in the Central Illustration.

**Figure:**
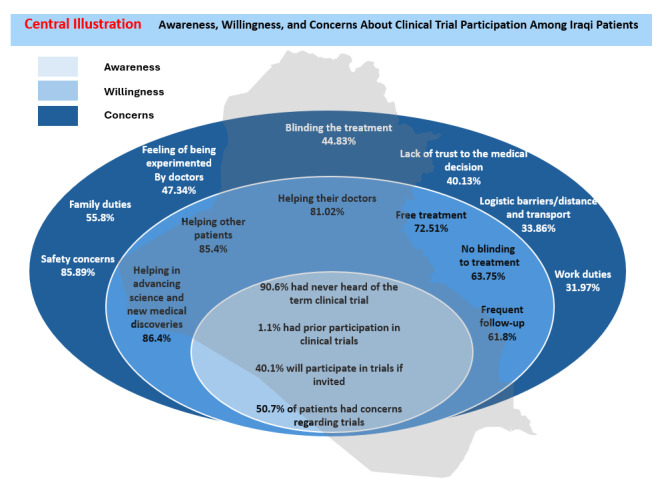


## Introduction

A clinical trial (CT) is a research study involving human participants, designed to evaluate the safety and effectiveness of medical interventions, such as drugs, surgical and radiological procedures, medical devices, behavioral therapies, preventive strategies, and modifications in care processes (1). These studies are governed by rigorous methodological standards, undergo meticulous planning and evaluation, and require approval by relevant regulatory and ethics authorities prior to initiation (1). CTs are widely regarded as the gold standard for generating high-quality evidence to inform clinical practice and guide healthcare policy. The primary motivators for participation include altruism, the desire to contribute to scientific advancement, potential personal health benefits, and occasionally, financial compensation (2,3). Despite their importance, CTs, like other research studies, remain underrepresented in many developing countries due to limited industry interest, a shortage of trained research personnel, logistical or bureaucratic barriers, and administrative challenges, such as increasingly complex ethics review processes, excessive paperwork, and sequential regulatory approvals (4,5). Despite their central role in evidence-based medicine, the global distribution of CTs remains profoundly inequitable. Low- and middle-income countries (LMICs), which account for the majority of the global disease burden, contribute disproportionately little to the design, conduct, and participant enrollment of randomized CTs. Multiple bibliometric and registry-based analyses demonstrate that most trials are conducted in high-income countries, with limited representation of populations from LMICs, raising concerns about the external validity, generalizability, and equity of resulting evidence. This imbalance is particularly consequential for chronic and non-communicable diseases, where genetic, environmental, and sociocultural factors may influence disease presentation, treatment response, and outcomes.

Developing countries have the heaviest burden of disease and thus offer substantial opportunities for research-driven impact, particularly in reducing early mortality and addressing neglected health priorities (5,6,7). Furthermore, developing countries provide a unique and valuable research environment, characterized by large treatment-naïve populations, higher disease prevalence, and more advanced stages of illness at presentation (8).

The Middle East and North Africa (MENA) region exemplifies these global disparities in clinical research participation. Despite a high and growing burden of cardiovascular disease, cancer, and metabolic disorders, countries in the MENA region collectively account for less than 1% of registered global CT sites. Existing studies from the region demonstrate substantial heterogeneity in research engagement, with relatively higher awareness and participation reported in countries such as Jordan and Saudi Arabia, while many countries affected by conflict or limited research infrastructure remain markedly underrepresented. Importantly, available data suggest that patient awareness, trust in medical research, and perceived risks vary widely across the region, underscoring the need for country-specific assessments rather than extrapolation from neighboring settings.

The MENA region is notably underrepresented in global clinical research, accounting for less than 1% of registered trials (9). Jordan stands out as the leading Arab country in terms of CT implementation and registration (8). In contrast, most global CTs are concentrated in high-income countries, with the United States, Germany, and France ranking as the top three hosts of trial sites (10). Globally, CT implementation faces numerous barriers, including low public awareness, pervasive misconceptions, time constraints, lack of trust in medical research, and concerns regarding experimentation and safety (11). These challenges are further complicated by insufficient health literacy and inadequate communication between researchers and potential participants (11). Addressing such issues requires strengthening the ethical and regulatory frameworks, simplifying consent processes, and fostering transparent, culturally sensitive researcher-participant engagement (12). Suboptimal recruitment in CTs can significantly affect CT validity by leading to underpowered studies, inconclusive results, non-generalizable findings, and increased costs (13). In oncology, for example, less than 5% of adult cancer patients participate in CTs, reflecting the cumulative effect of structural, clinical, socioeconomic, and psychological barriers (14). Health professionals often cite patient misconceptions and negative attitudes as significant obstacles to enrollment in randomized controlled trials (RCTs), particularly in cancer care (15). Multinational CTs can mitigate some of the recruitment challenges by enhancing participant diversity and increasing the generalizability of trial outcomes across various healthcare systems and populations (16). Yet a significant imbalance persists—diseases prevalent in high-income countries are evaluated approximately seven to eight times more often than those endemic to low- and middle-income regions. This disparity reflects the commercially driven priorities of pharmaceutical sponsors, many of whom design trials in developing countries to address questions relevant primarily to wealthier markets (17,18).

Although several studies from the Middle East have examined public and patient perceptions of CTs, the majority originate from countries with relatively well-established research infrastructures. In contrast, Iraq remains markedly underrepresented in global clinical research. Decades of armed conflict, health system disruption, and limited integration into international research networks have contributed to a severely underdeveloped CT ecosystem. While emerging evidence indicates growing interest among Iraqi healthcare professionals in conducting clinical research, substantial structural barriers persist, including inadequate research infrastructure, the absence of electronic health records, and limited formal training in clinical research methodologies (19). Critically, patient-level data on awareness, perceptions, and willingness to participate in CTs in Iraq are almost entirely lacking, severely constraining the development of ethically sound, culturally appropriate, and operationally feasible research initiatives.

Within this context, it is essential to examine the factors influencing CT participation in underrepresented populations. Iraq, as a setting with historically limited clinical research capacity, offers a uniquely informative context in which to explore these dynamics. This study seeks to examine Iraqi patients’ knowledge of CTs, their motivators and concerns, and their willingness to participate, thereby contributing to the sparse literature on patient-centered trial engagement in the MENA region.

Understanding patient perspectives is fundamental to ethical clinical research, particularly in LMICs, where limited health literacy, constrained access to information, and mistrust of medical research may undermine truly informed consent. Prior studies have consistently demonstrated that insufficient knowledge, heightened safety concerns, and poor communication between researchers and participants are among the most significant barriers to CT participation worldwide. Addressing these patient-level factors is therefore essential not only to improve recruitment but also to ensure that participation is voluntary, informed, and consistent with principles of justice, respect for persons, and ethical research conduct.

Accordingly, this study aims to systematically assess Iraqi patients’ awareness, motivators, concerns, and willingness regarding CT participation. By addressing a critical evidence gap, the findings are intended to inform the design of patient-centered, ethically robust clinical research strategies in Iraq and in other low-resource and post-conflict settings with similarly underdeveloped research infrastructures.

## Methods

This is the second phase of the IRAQ-PPI (IRAQ-Patient Public Involvement) Project, which was supported by the Iraqi Scientific Council of Cardiology (19). The investigators developed a 16-item paper form survey for targeted patients (20). The survey was developed in English language. The survey questions were designed by the investigators (ZAD, MP, and JL) after extensive literature review to assess what insights, barriers, and motivators were reported in prior studies especially in LMICs. Study investigators (ZAD, MP, and JL) revised the survey questions following feedback on their clarity and objectivity from all the investigators. The survey was completed by the investigators (SKH, RSN, ASA, MSS, MAQ, and MDM) during face-to-face interviews with the target population. The investigators received training and guidance from the principal investigator to standardize the interviews. Two external pilot phases were carried out, during which 20 patients were interviewed to evaluate the clarity of the survey questions. All feedback received was carefully reviewed, and necessary amendments were made to the survey accordingly. ZAD then designed the final paper form, which was approved by all investigators prior to use in the interviews.

The survey questions included: demographics of respondents, knowledge and insights of respondents toward CTs, and main motivators and concerns for participation in CTs. It is important to highlight that at the start of the survey, if a respondent reported lack of knowledge of the term ‘clinical trial,’ the investigator explained the meaning in simple Arabic to enable the respondent to participate.

Participants were adult and adolescent patients and patient attendants attending or admitted for any medical condition at five teaching hospitals in Iraq between October 2023 and February 2024. Recruitment was intentionally non-disease-specific. Eligible participants were approached consecutively during routine clinical care across five teaching hospitals to minimize selection bias and enhance sample heterogeneity. This approach was adopted to capture general patient perspectives on CT participation, irrespective of specific diagnoses or clinical categories.

This multi-center recruitment strategy was intended to improve representativeness across diverse patient groups within the Iraqi healthcare setting.

Inclusion criteria were the ability to communicate in Arabic and provide informed consent or assent; individuals unable to complete the interview were excluded.

Adolescents aged 15–17 years were eligible for participation, reflecting the study’s aim to capture general patient perspectives across age groups commonly engaged in healthcare decision-making. The survey was classified as minimal risk, as it involved a structured interview without any clinical intervention, alteration of care, or collection of biological samples.

For participants under 18 years of age, participation required dual consent procedures, including informed consent from a parent or legal guardian and assent from the adolescent participant. All information was provided in clear, age-appropriate Arabic, and participants were informed that participation was entirely voluntary and would not affect their medical care in any way. Adolescents were free to decline or withdraw at any point without consequence.

The inclusion of minors and the consent procedures were explicitly reviewed and approved by the institutional ethics committee. Only a small proportion of the total sample consisted of adolescents. These safeguards were implemented to ensure respect for autonomy, protection of vulnerable participants, and adherence to ethical standards for research involving minors.

The study was compliant with the national ethical protocols and ethical approval was obtained from Al-Kindy College of Medicine/University of Baghdad, with ethics number 194, dated at 22/06/2022.

### Statistical analysis

Descriptive statistics was used to analyze the survey responses. All frequencies were expressed as percentages. Categorial variables were compared using the Chi-square test.

Multivariable logistic regression was performed with overall willingness to participate (Yes = 1 vs. No/Don’t know = 0) as the dependent variable. Predictors entered simultaneously included age, sex, educational level (below college vs. college/postgraduate), awareness of the term ‘clinical trial,’ prior participation in a CT, concerns regarding CTs, willingness to participate in procedural trials, willingness to participate in educational/telemedicine/digital trials, willingness if drug safety was confirmed, and willingness if drug safety was unknown.

All predictors were entered simultaneously into the model to assess their independent associations with willingness after adjustment for potential confounding. Results are presented as odds ratios (ORs) with corresponding 95% confidence intervals (CIs). Trial-attribute variables were included intentionally to evaluate internal consistency of participant decision-making and to identify which trial characteristics most strongly influence overall willingness to participate, rather than to infer causal relationships. Statistical significance was set at a two-sided p-value < 0.05. IBM SPSS statistics version 26 was used for statistical analysis of participants’ characteristics with their responses in univariate and multivariate analyses.

## Results

The total number of respondents was 631 [379 (60.1%) were women]. The mean age was 41.7 ± 16.2 years; 13 participants were aged 15–17 years.

Highest education of respondents was reported as follows: 30.3% had primary school education and 14.4% no education, while a bachelor’s degree was attained by 18.1% (Table 1).

**Table 1:** Baseline characteristics of survey respondents.


BASELINE CHARACTERISTIC	NUMBER	PERCENTAGE

Age (Mean ± SD)	41.68 ± 16.19	

Female	379	60.1

Education*		

No education	91	14.4

Primary	191	30.3

Intermediate	108	17.1

High school	116	18.4

Bachelor	114	18.1

Master degree	7	1.1

PhD	3	0.5


*One patient had missing data regarding the education level.

**Figure 1:**
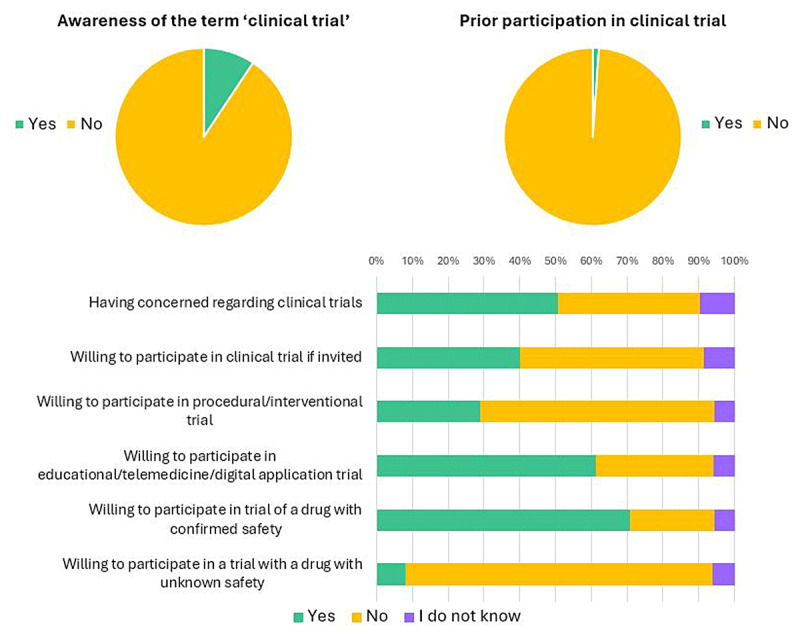
Awareness and insights of respondents towards clinical trials. Participants’ awareness of the term ‘clinical trial’ and prior experience with clinical trial participation, illustrating the proportion of participants who had previously heard of the term and those who had ever participated in a clinical trial. Insights regarding clinical trials and future willingness to participate also were reported.

When asking about their insights regarding clinical trials, 90.6% of respondents had never heard of the term ‘clinical trial,’ 1.1% had previously participated in a CT, and 50.71% of patients had concerns regarding CT. When asked if they would participate in a CT if invited, 51.3% said ‘No,’ 40.1% said ‘Yes,’ and 8.6% said ‘I do not know.’ When patients were asked if they would participate in future invasive CT (where the intervention is an invasive procedure or surgery), 65.5% said ‘No,’ 28.8% said ‘Yes’ while 5.7% said ‘I do not know.’ On the other hand, 61.2% of patients reported that they would participate in a CT if the intervention was either an educational program, telemedicine use or the use of digital applications. Interestingly, 70.8% of patients said they would participate in a CT if a drug had confirmed safety, while 85.9% said they would not participate in a CT if the drug had unknown safety (see Figure 1).

**Figure 2:**
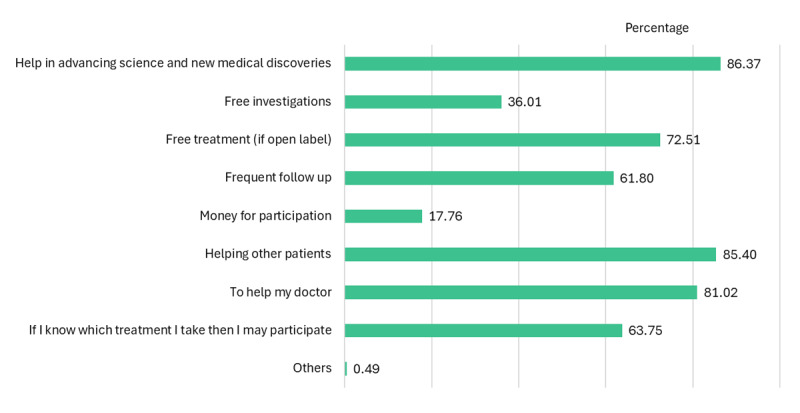
Motivators for participation in clinical trials. Self-reported motivators to participate in clinical trials reported mainly as help in advancing science and helping other patients.

When asking about motivators for participating in a CT, the main motivators were advancing science and new medical discoveries (86.4%) and helping other patients (85.4%) (see Figure 2).

**Figure 3:**
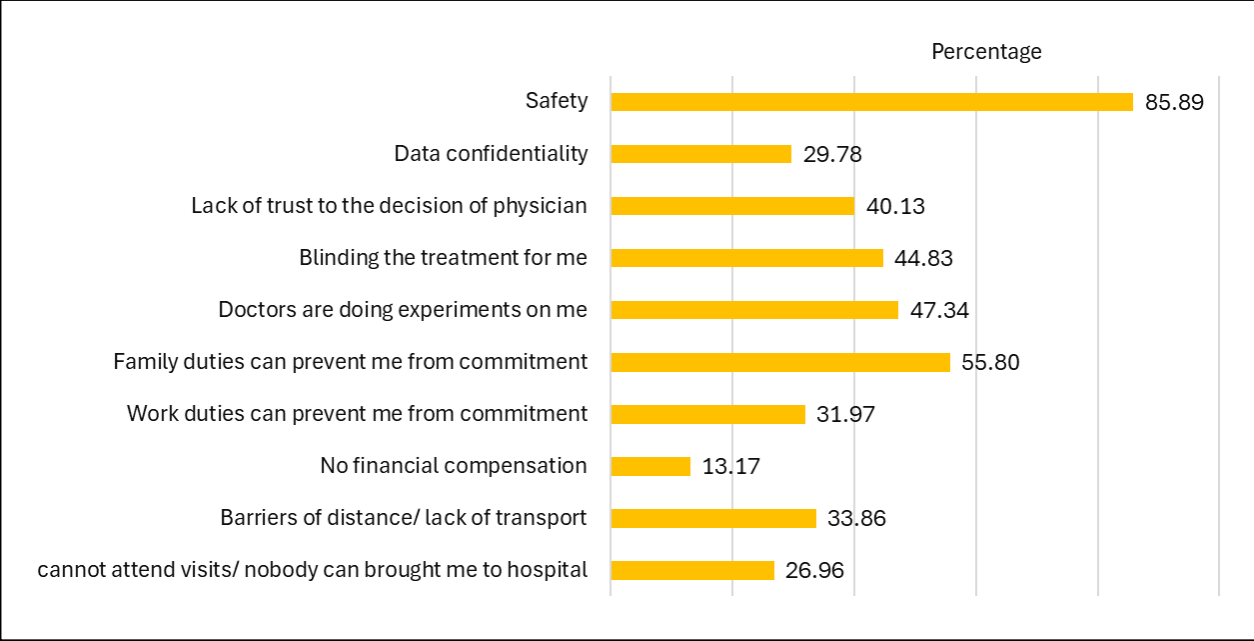
Barriers for participation in clinical trials. Self-reported barriers for participating in clinical trials are safety concerns and family commitments.

On further inquiry on the concerns of patients regarding CT, they reported that the main concerns were safety (85.9%), family duties that can prevent respondents from commitment to trial procedures (55.8%), and feeling the doctors are experimenting on them (47.34%) (see Figure 3).

**Figure 4:**
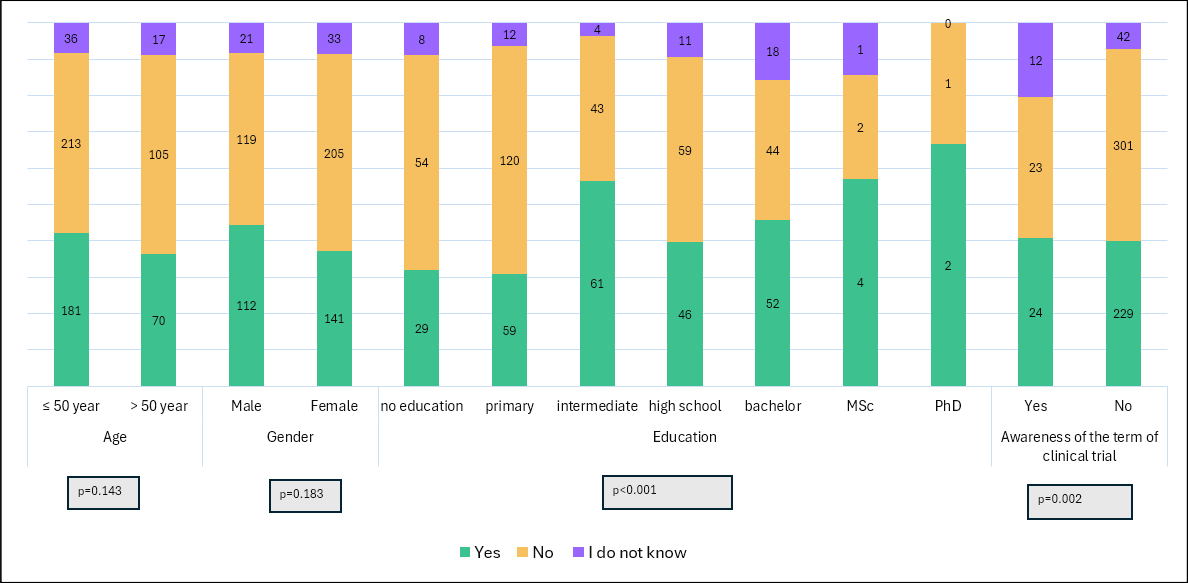
Willingness to participate in clinical trials according to respondents’ characteristics. Univariate analysis showed that main determinants of willingness to participate in future clinical trials are education and awareness of the term ‘clinical trials.’

Willingness to participate in future trials was significantly different according to the level of education. Willingness was higher in those with postgraduate degrees (57.14 % of MSc holders and 66.66% of PhD holders were willing to participate vs. 28.57% and 33.33% respectively, who indicated that they would not be willing to participate.) while 59.34% of those with no education and 62.82% of those with primary education were not willing to participate if invited (p < 0.001) (see Figure 4).

**Figure 5:**
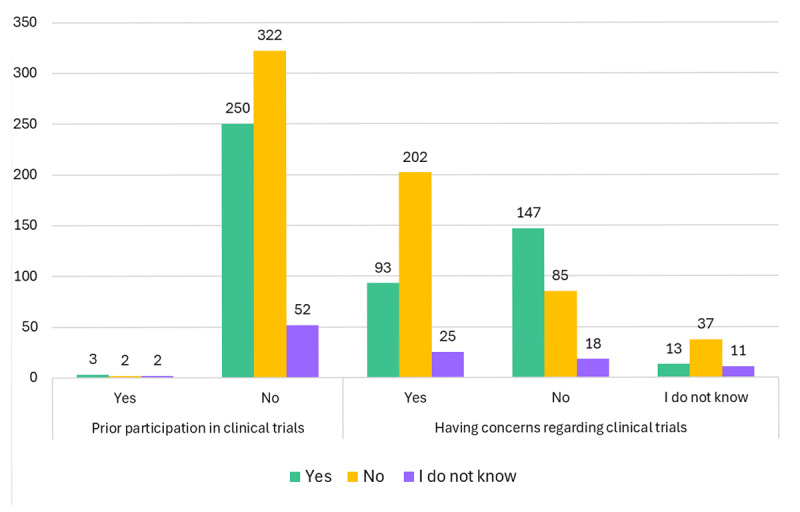
Willingness to participate in clinical trials according to prior trial participation and respondents concerns.

Knowledge of the term ‘clinical trial’ was also a determinant for the willingness of respondents to participate in a CT if invited. Among those who knew the term, 40.67% were willing to participate if invited while 38.98% were not willing. Among those who did not know the term ‘clinical trials,’ 40.03% were willing to participate while 52.62% were not (p = 0.002). Having concerns regarding CTs was another determinant of willingness to participate; 58.8% of those who have no concerns would participate in a CT if invited, while 29.06% of those who had concerns regarding CTs would participate if invited (p < 0.001) (see Figure 5).

In multivariable logistic regression, the model was statistically significant [χ²(10) = 334.282, p < 0.001] with good explanatory power (Nagelkerke R² = 0.562) and an overall classification accuracy of 79.2%. Higher education (college/postgraduate) was independently associated with greater willingness to participate (OR 2.06, 95% CI 1.15–3.68; p = 0.015), whereas reporting concerns about CTs was associated with reduced willingness (OR 0.42, 95% CI 0.27–0.66; p < 0.001). Trial-attribute perceptions were the strongest independent correlates: willingness to participate in procedural trials (OR 5.10, 95% CI 3.08–8.46; p < 0.001), willingness to participate in educational/telemedicine/digital trials (OR 5.10, 95% CI 2.90–8.99; p < 0.001), and willingness when drug safety was confirmed (OR 12.39, 95% CI 5.28–29.06; p < 0.001) were all strongly associated with overall willingness. Willingness when drug safety was unknown remained positively associated but weaker (OR 2.69, 95% CI 1.02–7.05; p = 0.045). Age category, sex, awareness of the term ‘clinical trial,’ and prior trial participation were not independently associated with willingness after adjustment (Table 2).

**Table 2:** Multivariable logistic regression for willingness to participate in clinical trials*.


PREDICTOR	ADJUSTED OR [EXP(B)]	95% CI	P-VALUE

Age > = 50 Years	0.776	0.475–1.266	0.310

Female (vs. male)	1.389	0.883–2.184	0.155

Higher education (college/postgraduate vs. below college)	2.057	1.150–3.682	0.015

Aware of the term ‘clinical trial’ (Yes vs. No)	0.923	0.432–1.975	0.837

Prior clinical trial participation (Yes vs. No)	0.733	0.082–6.558	0.781

Has concerns regarding clinical trials (Yes vs. No/Don’t know)	0.417	0.266–0.656	<0.001

Willing to participate in procedural trials (Yes vs. No/Don’t know)	5.104	3.079–8.459	<0.001

Willing to participate in educational/telemedicine/digital trials (Yes vs. No/Don’t know)	5.104	2.898–8.989	<0.001

Willing if drug safety is confirmed (Yes vs. No/Don’t know)	12.386	5.279–29.060	<0.001

Willing if drug safety is unknown (Yes vs. No/Don’t know)	2.686	1.024–7.045	0.045


*Model performance: χ²(10) = 334.282, p < 0.001; Nagelkerke R² = 0.562; overall classification accuracy = 79.2%.

## Discussion

CT participation is a cornerstone for generating evidence-based medical knowledge, yet global inequities in recruitment and therefore participation, persist, particularly in LMICs. These disparities threaten the generalizability of trial findings and create gaps in therapeutic advances and implementation and use of effective interventions. Iraq, despite bearing a substantial burden of different diseases remains underrepresented in CTs. Understanding population perceptions is essential because cultural, educational, and socioeconomic factors strongly influence participation decisions. Apart from a small-size survey (21), no data from Iraq explored patient views regarding participation in CTs. This study adds novel insights by being the first to systematically explore the awareness, attitudes, insights, and motivators of Iraqi patients toward CTs, highlighting critical areas for intervention to enhance recruitment and inclusivity in future research.

The striking finding in our work is that the majority of participants (90.6%) have never heard the term ‘clinical trial’ before, and only 1.1% participated in previous trials. More than half of the patients had concerns about CTs, with safety reported as primary concern, while helping in new medical discoveries and helping other patients were driving motivators of our participants’ willingness to participate in CTs in the future.

The lack of understanding about CT in our study is high compared to neighboring countries—taking Saudi Arabia as an example, when interviewed, only 9.1% of patients were unaware of CT (22). Additionally, a population-based survey conducted in Jordan in 2019 revealed that 74% of the population was aware of CTs (23). Multiple surveys from developed countries demonstrated a higher level of population awareness of CT, as in the French survey, where 64.8% were familiar with CTs and 72% had a positive attitude towards them (24). An Italian survey among health consumers revealed that 69% were aware about the existence of clinical research (25). Lower rates of awareness were noted in Lebanon and Oman at 45% and 31.3%, respectively (26,27). This lack of knowledge among Iraqi patients about clinical research potentially stems from multiple factors, including insufficient public health campaigns, general mistrust of medical institutions, and limited education about research. Furthermore, the sample that we studied had low educational attainment, with around 44% who had attained primary education or less.

Given the profound lack of awareness identified in this study, structured and pragmatic national awareness campaigns integrated into existing healthcare delivery are essential. These initiatives should leverage trusted healthcare professionals as key messengers and utilize culturally adapted communication strategies, including social media, community outreach, and patient education materials in local dialects. Furthermore, establishing simplified, transparent consent processes and enhancing ethics committee efficiency could reduce administrative barriers that currently hurdle clinical research in Iraq.

This study uncovers main motivations that drive individuals to participate in CTs, revealing a deep-seated desire to contribute to new medical discoveries and help other patients in need. Despite Iraq being a developing country with a high unemployment rate and economic instability, financial compensation emerged as the least compelling incentive for participation (only for 17.76% of participants). These findings align with multiple systematic reviews from countries of the region, including Jordan and the Eastern Mediterranean, illustrating a common thread of altruism (23,28). Moreover, they reflect motivations found in wealthier neighboring countries such as, Saudi Arabia, Qatar, and UAE, where the focus remains on noble contributions rather than monetary gain (22,29,30). Other studies highlight different motivators: for instance, an Indian systematic review identified personal health benefits and physicians’ trust as key reasons for participation (31). In Egypt, a pilot study similarly found that personal health benefits are the main motivator (32). A narrative review of factors affecting trial participation in Western countries demonstrated that personal benefit is the key motivating factor (33). A systematic review of 13 studies (six from the USA, six from Europe, and one from Malawi) encompassing over 2,000 healthy volunteers found that while financial compensation was the primary motivation for participation in CTs, other commonly reported motivations included contributing to scientific advancement or public health, gaining access to healthcare benefits, personal interest in the study’s objectives, curiosity, and opportunities for social interaction (34). This trend was also observed in Brazil, where financial gain and the availability of therapeutic alternatives were significant motivators (35).

The common reasons for refusal to participate in CTs in our survey are safety (85%), family duties affecting commitment to trial procedures (55.8%), and fear of being experimented upon; the least reported concern is a lack of financial compensation. A population-based survey in the Arab MENA region identified several common barriers such as time constraints, fear of research procedures, and a lack of interest (23). Similarly, several studies in countries of the region, such as Qatar and Kuwait, reported time constraints and fear as prevalent barriers (29,36). In Saudi Arabia, cancer patients highlighted the fear of side effects (82.6%) and insufficient information as common obstacles (37). In a meta-analysis, Indian participants expressed mistrust in trial organization, concerns about the safety of the trial, and psychological factors as reasons hindering their involvement (31). A survey in Malaysia revealed that being female, unemployed, and poor knowledge were linked to declining participation in clinical research (38). Another meta-analysis found that among patients of Chinese heritage, mistrust secondary to a lack of knowledge and understanding of CT was the primary reason for refusal to participate (39). In the USA, common reasons for refusal to participate in cardiovascular CTs included older age, female sex, and out-of-state residence (40). In our study, 65.5% reported that they would refuse to participate in a CT if the study involved invasive interventions. This is consistent with other studies that considered study design and the method of data collection as key factors that serve both as a motivator and a barrier. Specifically, patients are willing to participate in observational clinical research, such as those involving questionnaires, and studies with minimally invasive procedures; however, they are often reluctant to take part in drug trials and studies with invasive procedures, such as tissue biopsies (41,42,43).

The disparity in the motivators and barriers for participation across different countries can be attributed to various factors, including individual characteristics, cultural beliefs, socioeconomic factors, and health care system attributes, all of which influence individuals’ decisions to participate. The unique sociocultural landscape of Iraq, characterized by strong family obligations and limited trust in medical research, exaggerate the existent barriers like concerns over time commitment and safety. Unlike Western populations, where personal benefit and financial incentives often dominate decision-making, Iraqi patients demonstrated altruistic motivations as primary drivers. These findings advocate culturally tailored recruitment strategies, emphasizing community benefit, physician trust-building, and family engagement as opposed to predominantly monetary incentives.

The barriers also expose an underlying ethical dimension; if most patients lack basic understanding of trials and harbor safety fears, achieving truly informed consent becomes questionable. This raises concerns about the ethical conduct of global trials in LMICs, where participants might agree without a clear grasp of the risks. Stronger ethical oversight, transparent communication, and community engagement frameworks are critical to ensure equity and protect vulnerable populations.

Our study shows that less than half (40.1%) of the Iraqi patients would agree to participate in CTs if invited. This figure is lower than that in Jordan, where 63% have the desire to participate in CTs if invited (23). Interestingly, over 60% of respondents expressed willingness to participate in trials involving digital tools, such as telemedicine and educational interventions. This indicates a promising pathway for hybrid and decentralized trial models in Iraq, which could mitigate logistical barriers, reduce patient burden, and foster broader engagement. Future research should explore digital literacy levels and infrastructure readiness to capitalize on this opportunity.

In the multivariable logistic regression, educational attainment and trial-related perceptions, not basic demographics, emerged as the dominant independent determinants of willingness. Specifically, higher education (college/postgraduate) remained independently associated with increased willingness, whereas the presence of concerns remained independently associated with reduced willingness, indicating that structural (education-related) and perceptual (fear- and trust-related) barriers persist even after adjustment.

This pattern suggests that education likely acts as a proxy for health literacy, prior exposure to research concepts, and perceived self-efficacy in navigating consent and trial procedures, highlighting the need for trial communication strategies that are effective for participants with limited formal education.

In contrast, willingness to participate increased markedly when participants endorsed specific trial scenarios—particularly procedural trials, educational/telemedicine/digital trials, and most strongly when drug safety was confirmed, thus highlighting the central role of trial-attributes and safety assurances in participation in decision-making. These trial-attribute variables should be interpreted as indicators of participant preferences and internal decision consistency rather than as independent causal predictors, as they reflect context-specific expressions of overall willingness that are conceptually embedded within the outcome itself.

Although overall willingness to participate in trials involving invasive or procedural interventions was low at the descriptive level, endorsement of procedural trial participation emerged as a strong independent correlate of overall willingness in multivariable analysis. This finding should not be interpreted as procedural burden increasing willingness; rather, it reflects the presence of a small but highly motivated subgroup of participants who remain willing to participate even under more demanding trial conditions. In this context, willingness to engage in procedural trials functions as an indicator of decisional robustness and commitment rather than a causal determinant of participation. These results highlight meaningful heterogeneity in patient preferences and suggest that recruitment strategies may benefit from identifying and engaging such trial-ready subgroups, while simultaneously addressing procedural concerns among the broader population.

Although awareness of the term ‘clinical trial’ was associated with willingness in univariate analysis, it did not remain an independent predictor after adjustment, suggesting that awareness alone may be insufficient without addressing concerns and communicating safeguard measures. These patterns are consistent with regional evidence showing associations between education and participation-related attitudes (e.g., Saudi Arabia) (37,44) and with broader findings across Asian and European populations where educational attainment and perceptions of risk/trust shape enrollment decisions (45,46). Similarly, European surveys have linked attitudes toward CTs with familiarity and trust in researchers and institutions (24,25).

Only 1.1% of participants had previously enrolled in a CT, with similarly low rates reported in other Arab countries of the region, including Oman, Qatar, and Saudi Arabia, where previous participation rates were 6.5%, 5.7%, and 5.5%, respectively (27,29,37). These figures are significantly lower than those found in developed countries, such as England and Romania, where the rates are 30.1% and 23%, respectively (47,48). Additionally, an international survey involving 12,427 participants from 68 countries demonstrated that 17.7% of the population had previous participation in clinical research studies (49).

These findings underscore an urgent need for global sponsors and policymakers to prioritize LMIC populations in trial recruitment strategies. Limited awareness and high safety concerns, as observed in Iraq, likely mirror challenges across other LMICs, making this issue not just local but globally relevant. Underrepresentation of these populations in trials risks creating therapies optimized for high-income settings while overlooking diverse genetic, environmental, and cultural contexts present in LMICs. Thus, addressing these disparities is not merely an ethical obligation but a scientific imperative.

The IRAQ-PPI Project represents a pioneering, multi-phase initiative to establish a robust CT ecosystem in Iraq by systematically engaging both cardiovascular healthcare professionals and patients (19,20). Phase 1 surveyed 255 cardiovascular professionals and revealed enthusiastic support for conducting CTs in Iraq (95.7%). Key barriers included lack of infrastructure, electronic health records, and clinician research literacy, while motivating factors were the introduction of electronic health systems and targeted population and provider training (19).

Building upon these foundational insights, Phase 2 (the current patient-focused study) offers the first comprehensive assessment of Iraqi patients’ perceptions (20). It highlights severe knowledge deficits, prominent safety and familial concerns, and strong altruistic motivations to contribute to advancing science and helping others. Notably, willingness to participate is most strongly shaped by educational attainment, trial-related concerns, and perceived safety, alongside a clear openness to non-invasive and digital trial models. Looking ahead, Phase 3 envisions the creation of Iraq’s first PPI nucleus through an intensive educational program in collaboration with the Iraqi Scientific Council of Cardiology (19). This phase aims to co-create awareness, research literacy, and PPI tools, while mobilizing medical students and junior clinicians as a sustainable cardiovascular research workforce to transform trial culture and infrastructure.

Together, these phases present a roadmap for transformative change in Iraq’s clinical research landscape, from understanding foundational barriers among healthcare professionals, to mapping patient attitudes, and ultimately building community-driven education and involvement frameworks. This integrated approach holds the promise of not only improving trial recruitment and diversity but also advancing global equity and ensuring that medical interventions are effective and representative of populations in low- and middle-income settings.

This study represents the first systematic exploration of Iraqi patients’ perceptions toward CTs, addressing a major gap in evidence from LMICs that are disproportionately affected by non-communicable diseases yet remain underrepresented in global research. The large sample size, inclusion of five major teaching hospitals, and face-to-face interview design strengthened the reliability of responses and minimized literacy-related barriers to survey completion. The study also provides unique insights into both motivators and concerns to participation, offering actionable directions for future interventions and educational strategies. However, the study has several limitations. The recruitment from hospital-based populations may not fully reflect community perspectives, especially those of rural or marginalized groups. Self-reported responses may have introduced social desirability bias, particularly regarding willingness to participate. In addition, the survey instrument, while validated, was limited to 16 items and may not have captured the full complexity of cultural and socioeconomic influences on trial participation. It would be valuable to conduct qualitative interviews in the future to get a deeper understanding of how people think about CTs. Furthermore, we did not ask what health problems the respondents had; there may be certain disease groups who are more willing to participate in trials (e.g., people usually become more willing if their disease is terminal). Finally, generalizability to other LMICs should be made with caution, as findings are context-specific to Iraq.

### Recommendations and future directions

**Figure 6:**
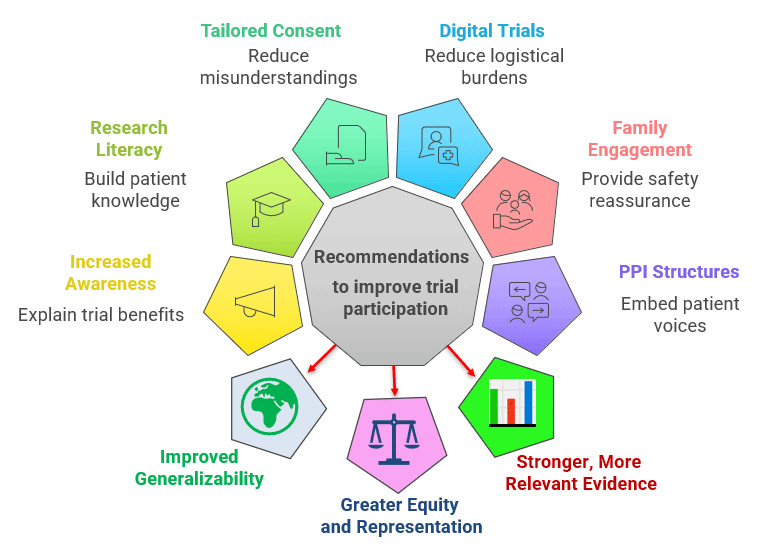
Patient-centered strategies and expected benefits to improve clinical trial participation in LMICs: improving patient-centered trial recruitment strategies in LMICs can enhance the generalizability, equity, and real-world relevance of clinical research evidence.

Improving CT participation in Iraq and similar low- and middle-income settings requires patient-centered strategies that address awareness, safety concerns, cultural factors, and trial design preferences. As summarized in Figure 6, implementing these approaches can enhance the generalizability, equity, and real-world relevance of CT evidence by ensuring broader inclusion of underrepresented populations.

## Conclusions

Iraqi patients demonstrate substantial gaps in awareness of CTs alongside significant safety concerns, yet show encouraging altruism and openness to less burdensome research models. Strengthening public education, improving transparency around trial safety, and adopting patient-centered designs are essential to building trust and enhancing participation. Advancing engagement in underrepresented settings such as Iraq is critical to ensure that global clinical research reflects the populations it seeks to serve.

## Data Accessibility Statement

Data are available from the corresponding author upon reasonable request.
